# The Prognostic Value of Lymphovascular Invasion in Patients With Upper Tract Urinary Carcinoma After Surgery: An Updated Systematic Review and Meta-Analysis

**DOI:** 10.3389/fonc.2020.00487

**Published:** 2020-04-22

**Authors:** Lijin Zhang, Bin Wu, Zhenlei Zha, Hu Zhao, Jun Yuan, Yejun Feng

**Affiliations:** Department of Urology, Affiliated Jiang-yin Hospital of the Southeast University Medical College, Jiangyin, China

**Keywords:** lymphovascular invasion, upper tract urinary carcinoma, radical nephroureterectomy, prognosis, meta-analysis

## Abstract

**Background and Purpose:** Although the prognostic value of lymphovascular invasion (LVI) for upper tract urinary carcinoma (UTUC) has been reported, there is a lack of consensus regarding the prognostic factor of LVI in UTUC after radical nephroureterectomy (RNU). The aim of the present study was to evaluate the contemporary role of LVI using systematic review and meta-analysis.

**Materials and Methods:** Using Preferred Reporting Items for Systematic Reviews and Meta-Analysis guidelines, we performed a systematic search of Web of Science, PubMed, and EMBASE for all reports published up to July 2019. Cumulative analyses of hazard ratios (HRs)/odds ratios (ORs) and their corresponding 95% confidence intervals were conducted to assess the association between LVI and oncological outcomes and clinicopathological features.

**Results:** Our meta-analysis included 31 eligible studies containing 14,653 patients with UTUC (81–1,363 per study). Our results indicated a significant correlation of LVI with worse cancer-specific survival (HR = 1.59, *p* < 0.001), overall survival (HR = 1.55, *p* < 0.001), recurrence-free survival (HR = 1.46, *p* < 0.001), cancer-specific mortality (HR = 1.25, *p* = 0.047), and recurrence (HR = 1.23, *p* = 0.026). LVI was also correlated with advanced tumor stage (III/IV vs. I/II: OR = 7.63, *p* < 0.001), higher tumor grade (3 vs. 1/2: OR = 5.61, *p* < 0.001), lymph node metastasis (yes vs. no: OR = 4.95, *p* < 0.001), carcinoma *in situ* (yes vs. no: OR = 1.92, *p* < 0.001), and positive surgical margin (yes vs. no: OR = 4.38, *p* < 0.001), but not related to gender (male vs. female: OR = 0.98, *p* = 0.825), and multifocality (multifocal vs. unifocal: OR = 1.09, *p* = 0.555). The funnel plot test indicated no significant publication bias.

**Conclusions:** This study demonstrated that LVI was associated with aggressive clinicopathological features. LVI may serve as a poor prognostic factor for patients with UTUC after RNU.

## Introduction

The upper tract urothelial carcinoma (UTUC), which accounts for ~5% of all urothelial carcinoma, develops from the urothelium that lines the renal pelvis and the ureter (1). Although UTUCs share many similarities with bladder cancer, little is known about their pathogenesis, given the rarity of the disease. Radical nephroureterectomy (RNU) with bladder cuff excision is the gold standard curative therapy for localized UTUC; however, about 33% of patients with RNU will experience early tumor recurrence within 5 years (2), and the 5-years cancer-specific survival (CSS) is <50% for patients with early-stage UTUC (3). The current predictive nomograms based on preoperative parameters may guide surgeons for decision-making regarding RNU with or without lymphadenectomy (4). However, predicting oncologic outcomes is another major concern in patients with UTUC. Because there are aggressive features characteristic to UTUC, a comprehensive recognition of the potential prognostic factors for survival is critical.

Lymphovascular invasion (LVI), which is defined as the presence of tumor cells within lymphatic or vascular channels, is a significant step in tumor distant metastasis (5, 6). According to the recommended European urology guidelines, LVI is an independent prognostic factor for bladder cancer using cystectomy specimens (7). In 2013, Ku et al. (8) performed a meta-analysis of 17 studies and confirmed the significant prognostic role of LVI in RNU specimens. However, much of the raw data in the included literature were lost in their paper. No additional study was conducted to determine the relationship between LVI and other clinicopathological features. In recent years, many studies have contributed relevant information toward the clinicopathological implications of LVI. The purpose of this study was to investigate the relationship between LVI and the clinical outcomes in patients with UTUC to enhance our understanding of prognostic values of LVI and facilitate efficient and prompt clinical decision-making for the patient.

## Materials and Methods

### Literature Search Strategy

Using Preferred Reporting Items for Systematic Review and Meta-analysis (PRISMA) guidelines (9), we (Z.L.Z. and H.Z.) conducted a computerized search using PubMed, EMBASE, and Web of Science in July 2019 to identify studies that documented the incidence of LVI in patients with UTUC undergoing RNU. The combination of the following keywords were used: (“upper urinary tract tumor” OR “renal pelvis” OR “ureter”) AND (“radical nephroureterectomy”) AND (“lymphovascular invasion”) AND (“prognosis” OR “clinical outcome” OR “survival”). The language was restricted to English. At the same time, we manually screened the reference lists of the selected papers, including all of the relevant studies and reviews. For the data obtained from the published studies, no ethical approval and informed consent were required.

### Study Inclusion and Exclusion Criteria

The following inclusion criteria were used to select eligible studies: (a) the diagnoses of UTUC and LVI were pathologically confirmed; (b) treatment was limited to RNU; (c) the prognostic values [hazard ratios (HRs) and 95% confidence intervals (95% CIs)] of LVI for overall survival (OS), CSS, recurrence-free survival (RFS), cancer-specific mortality (CSM), and recurrence risk were reported. Accordingly, the exclusion criteria of the meta-analysis were as follows: (a) studies that were not written in English; (b) meeting abstracts, reviews, review papers, or case reports; and (c) no sufficient data to estimate the HRs and 95% CIs. If more than one article from one patient cohort was identified, the most complete article was selected.

### Data Extraction

Two authors (J.Y. and Y.J.F.) independently extracted data from the included studies using a predefined data extraction form. Discrepancies were resolved through discussion by a third author (B.W.). The following variables were recorded: patients' characteristics (first author's name, year of publication, geographical region, number of patients, ages, gender, study period, and follow-up duration), tumor characteristics (TNM stage, tumor grade, LVI, lymph node metastasis, tumor multifocality, tumor necrosis, and positive surgical margin), and outcomes of interest. Our primary outcomes included OS, CSS, RFS, CSM, and recurrence. When multivariate analysis and univariate analysis results were both presented in one study, we chose the multivariate analysis results because they account for confounding factors and are more accurate.

### Quality Assessment

The Newcastle Ottawa Scale (NOS) (10), which was recommended for evaluating non-randomized studies, was used to assess the quality of the selected studies. This scale assesses risk in three domains: patient selection, comparability of control and intervention groups, and assessment of outcomes. A score of 0–9 stars was allocated to each study. We defined high quality as a score of 6–9 and low quality as a score of <6.

### Statistical Analysis

Effect measures for the outcomes of OS, CSS, RFS, CSM, and recurrence were HRs and 95% CIs extracted from the published studies. We studied the associations between LVI and clinicopathological parameters of UTUC. The numbers of events were obtained from the original studies, and the odds ratios (ORs) and the corresponding 95% CIs were calculated. The heterogeneity across studies was tested by using Cochran's Q test and Higgins ***I***-squared statistic. There was marked heterogeneity if *P* ≤ 0.10 and/or *I*^2^ was >50%. A random-effects (RE) model was applied to pool results under significant heterogeneity; otherwise, a fixed-effects (FE) model was applied. A pooled HR ≥ 1 indicated poor survival for patients with an LVI expression. The source for interstudy heterogeneity was explored using subgroup analysis. Publication bias was evaluated by assessing the asymmetry of the funnel plot. Furthermore, Egger's test for funnel plots, which provides quantitative evidence, was employed to search for publication bias between the studies. To examine the stability and the reliability of the overall meta-analysis results, we performed the sensitivity analysis by excluding one study in turn. The statistical analyses were performed using Stata 12.0 software (Stat Corp, College Station, TX, USA). All *P*-values were two-sided, and *P* < 0.05 was considered to be statistically significant.

## Results

### Search Results

**Figure 1:**
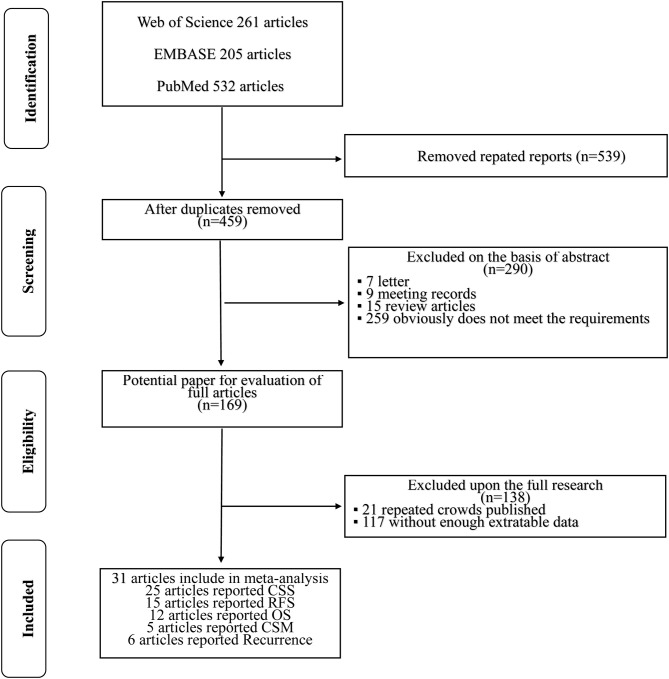
PRISMA flow chart of literature search and selection process.

The initial search yielded 998 references, and 539 studies were excluded because of duplication. After title and/or abstracts were screened, 169 articles remained for full-text assessment, and 290 articles were excluded, including reviews, letters, meeting abstracts, and other articles irrelevant to our study. In accordance with the study inclusion criteria, 138 articles were excluded for repeated crowds or without enough extractable data. Finally, 31 studies, which were retrospective in design, were included in this meta-analysis. A flow diagram about the literature search and study selection process is presented in Figure 1.

### Features of Included Studies

The summary characteristics of these studies are shown in Table 1. A total of 14,653 patients with UTUC (range, 81–1,363) were included in the study. The median or mean age of patients ranged from 62 years to 71 years. The 31 included articles were published from 2009 to 2019. Geographically, 20 studies were conducted in Asia, 7 in multicenters worldwide, 2 in the USA, 1 in France, and 1 in Serbia. All of the patients had received RNU as their primary treatment for UTUC. Of these studies, 12 studies reported OS, 25 studies reported CSS, 15 studies reported RFS, 5 studies reported CSM, and 6 studies reported recurrence. The characteristics of tumor features and pathologic outcomes are summarized in Table 2. LVI was detected in 24.8% (3,635/14,653) of pathological specimens of the included patients. According to the NOS, we assessed the quality of the 31 eligible studies (11–41). The quality scores of the studies varied from 7 to 9, with a mean of 8.7; therefore, all of the studies were of high quality (Supplementary Table 1).

**Table 1:** Clinical characteristics of the included studies in this meta-analysis.

**References**	**Country**	**Recruitment period**	**No. of patients**	**Age (years)**	**Gender (m/f)**	**Pelvicalyceal/ureteral/both**	**Follow-up (months)**	**Survival analysis**
Liu et al. (11)	China	2005–2013	180	Median (range) 67.2 (39–87)	109/71	NA	Median (range) 45.4 (3–180)	RFS, CSS
Li et al. (12)	China	1999–2015	885	Mean ± SD 66.9 ± 10.6	396/489	474/411	Median (IQR) 61 (38–102)	CSS, OS
Jan et al. (13)	China	2007–2017	424	Median (range) 70 (29–96)	189/235	191/138/95	Median (IQR) 35 (14–60)	CSS, OS
Aydin et al. (14)	Muti–centers	1990–2008	348	Median (IQR) 70 (64–77)	163/185	267/81	Median 36	RFS, CSS, OS
Tan et al. (15)	China	2003–2015	620	Mean ± SD 65.7 ± 11.3	355/265	350/161/109	Median (range) 51 (1–168)	RFS, CSS, OS
Kohada et al. (16)	Japan	1999–2016	148	Median (IQR) 71 (64–78)	112/36	82/66	Median (IQR) 35.5 (12–66)	RFS, CSS
Abe et al. (17)	Japan	2000–2015	214	Median (range) 70.5 (35–93)	151/63	127/82/5	Median (IQR) 41 (21–71)	RFS, CSS, OS
Nakagawa et al. (18)	Japan	1996–2013	109	Median (IQR) 71 (64–77)	67/42	50/23/36	Median (IQR) 46.5 (23.2–76.7)	RFS, CSS
Inokuchi et al. (19)	Japan	2005–2011	823	Median (IQR) 71 (63–77)	578/245	434/375/14	Median (IQR) 59.8 (23.3–66.2)	CSS, OS
Ikeda et al. (20)	Japan	1985–2013	399	Median (IQR) 67 (62–75)	307/92	213/186	Median (IQR) 43 (17–89)	RFS, CSS
Fan et al. (21)	China	2002–2013	101	Median 69	61/40	55/43/3	Median (range) 41.3 (4.2–106.5)	RFS, CSS
Cho et al. (22)	Korea	2004–2015	1,049	Median (IQR) 68.5 (60.5–74.3)	759/290	489/462/252	Median (IQR) 40 (18.4–64.8)	RFS, CSS, OS
Abufaraj et al. (23)	Muti–centers	1990–2008	678	Median (IQR) 69 (63–76)	380/298	478/200	Median (IQR) 37.5 (20–66)	Recurrence, CSM
Yan et al. (24)	China	2002–2012	795	NA	462/333	497/187/111	Median (IQR) 32 (17–60)	RFS, CSS, OS
Kobayashi et al. (25)	Japan	1990–2011	839	Median (IQR) 70.4 (63–78)	610/229	NA	Median (IQR) 34 (17–63)	Recurrence, CSS
Kang et al. (26)	Korea	2004–2014	566	Median (IQR) 70 (62–75)	401/165	258/308	Median) 33.8	RFS, CSS, OS
Fukushima et al. (27)	Japan	2001–2015	81	Median (range) 71 (41–87)	53/28	36/31/14	Median (range) 41 (4–170)	CSS, OS
Mathieu et al. (28)	Muti–centers	1990–2008	732	Median (IQR) 70 (63–76)	414/318	518/214	Median (range) 35 (16–64)	RFS,CSS
Lee et al. (29)	Korea	1986–2013	344	Mean ± SD 65.1 ± 10.6	240/104	146/147/51	Median (range) 53.9 (1–297)	CSS, OS
Lee et al. (30)	China	2004–2010	250	NA	108/142	129/122	Median 41	CSS
Park et al. (31)	Korea	1991–2010	392	Median (range) 64 (29–86)	299/93	NA	Median (range) 47.6 (2–257)	RFS, CSS
Lee et al. (32)	Muti–centers	1991–2008	622	Median (IQR) 69 (63–76)	346/276	452/170	Median (IQR) 27 (12–53)	Recurrence, CSM
Krabbe et al. (33)	USA	2000–2012	122	Median (range) 69 (35–92)	77/45	88/34	Median (range) 32 (1–149)	CSS
Kluth et al. (34)	Muti–centers	1975–2012	242	Median (IQR) 70 (63–77)	175/67	145/83/11	Median 9	CSM
Liu et al. (35)	China	1999–2010	421	Median (IQR) 62 (51–70)	285/136	225/196	NA	CSS
Hurel et al. (36)	France	1995–2010	551	Median (IQR) 69.4 (61.8–76.4)	365/188	302/169/80	Median (IQR) 26.8 (10.3–48.7)	RFS, CSS
Milojevic et al. (37)	Serbia	1999–2009	133	Mean ± SD 66.7 ± 8.9	77/56	88/45	Median (range) 35 (2–113)	Recurrence, CSS
Godfrey et al. (38)	USA	1990–2010	222	Mean ± SD 70 ± 11.4	124/87	170/41	Median (IQR) 27 (11–65.5)	OS
Novara et al. (39)	Muti–centers	1987–2008	762	Median (IQR) 68 (61–75)	527/235	401/232/48	Median (IQR) 34 (15–65)	Recurrence, CSM
Kim et al. (40)	Korea	1986–2006	238	Median (range) 64.1 (25–91)	164/74	134/104	Median (range) 64.1 (25–91)	RFS, CSS
Margulis et al. (41)	Muti–centers	1992–2006	1,363	Mean ± SD 69.7 ± 11.1	921/442	878/463/22	Median (range) 37.2 (1.2–250)	Recurrence, CSM

*m/f:male/femal; SD: standard deviation; NA, data not applicable; CSS: cancer-specific survival; OS: overall survival; RFS:recurrence-free survival; CSM: cancer-specific mortality*.

**Table 2:** Tumor characteristics of the included studies in this meta-analysis.

**Study**	**Staging system**	**Grading system**	**LVI +/ LVI -**	**Stage 1-2/ 3-4**	**Grade 1-2/ 3**	**LNM-/ LNM+**	**Unifocal/ Multifocal**	**Papillary/ Sessile**	**TN+/ TN-**	**PSM+/ PSM-**
Liu et al. (11)	2008 AJCC	2016 WHO/ ISUP	28/152	115/65	91/89	169/11	173/7	NA	7/173	NA
Li et al. (12)	2002 AJCC	1973 WHO/ ISUP	46/839	623/262	518/367	823/62	NA	771/114	114/771	NA
Jan et al. (13)	2009 AJCC	2004 WHO/ ISUP	115/299	244/180	22/402	399/25	308/116	97/278	86/338	NA
Aydin et al. (14)	2002 AJCC	1998 WHO/ ISUP	98/250	191/157	NA	314/34	270/78	286/62	62/286	NA
Tan et al. (15)	2002 AJCC	WHO/ ISUP	100/520	310/310	158/462	554/62	517/103	193/427	NA	50/570
Kohada et al. (16)	2002 AJCC	1998 WHO/ ISUP	55/93	82/66	60/88	140/8	148/0	NA	NA	12/136
Abe et al. (17)	2002 AJCC	1973 WHO/ ISUP	96/118	121/83	101/113	195/19	209/5	NA	NA	11/203
Nakagawa et al. (18)	2009 AJCC	2004 WHO/ ISUP	78/31	0/109	40/69	21/88	73/36	NA	NA	9/100
Inokuchi et al. (19)	2002 AJCC	NA	252/52	459/324	444/379	787/26	809/14	NA	NA	34/789
Ikeda et al. (20)	2002 AJCC	1973 WHO/ ISUP	138/236	237/162	285/109	359/40	399/0	NA	NA	32/358
Fan et al. (21)	2002 AJCC	1998 WHO/ ISUP	14/87	47/54	25/76	92/9	91/10	60/31	NA	NA
Cho et al. (22)	2009 AJCC	1998 WHO/ ISUP	202/847	623/426	304/705	965/84	889/160	NA	NA	NA
Abufaraj et al. (23)	2002 AJCC	1973 WHO/ ISUP	135/543	452/226	174/504	629/49	533/145	558/120	597/81	NA
Yan et al. (24)	2010 AJCC	1998 WHO/ ISUP	169/626	390/405	212/583	711/84	684/111	256/539	NA	76/719
Kobayashi et al. (25)	AJCC	1973 WHO/ ISUP	326/513	415/424	347/492	783/56	715/124	NA	NA	NA
Kang et al. (26)	AJCC	1998 WHO/ ISUP	119/447	346/220	178/388	NA	517/49	NA	NA	NA
Fukushima et al. (27)	2002 AJCC	1973 WHO/ ISUP	50/31	37/44	31/50	74/7	67/14	NA	NA	NA
Mathieu et al. (28)	2002 AJCC	1998 WHO/ ISUP	153/579	480/252	187/454	677/55	577/155	601/131	97/635	NA
Lee et al. (29)	2010 AJCC	1998 WHO/ ISUP	86/258	144/200	53/291	265/79	293/51	NA	NA	NA
Lee et al. (30)	AJCC	2004 WHO/ ISUP	60/190	166/84	57/193	232/18	191/59	NA	NA	NA
Park et al. (31)	1997 AJCC	1973 WHO/ ISUP	89/303	248/144	196/196	357/35	NA	265/127	NA	25/367
Lee et al. (32)	2002 AJCC	2004 WHO/ ISUP	140/482	396/226	164/458	569/53	498/124	518/104	85/537	NA
Krabbe et al. (33)	2010 AJCC	NA	28/94	87/35	27/95	113/9	63/59	80/42	NA	NA
Kluth et al. (34)	2010 AJCC	2004 WHO/ ISUP	131/111	76/166	NA	191/51	139/60	83/47	70/159	NA
Liu et al. (35)	2002 AJCC	1998 WHO/ ISUP	101/320	248/173	215/206	325/96	288/133	NA	NA	36/385
Hurel et al. (36)	2009 AJCC	1973 WHO/ ISUP	163/388	266/246	331/415	504/47	471/80	NA	NA	53/498
Milojevic et al. (37)	1997 AJCC	1998 WHO/ ISUP	78/55	47/86	46/87	128/5	86/47	NA	NA	NA
Godfrey et al. (38)	2010 AJCC	1998 WHO/ ISUP	68/143	137/74	77/134	197/14	NA	NA	NA	18/193
Novara et al. (39)	2002 AJCC	1973 WHO/ ISUP	148/614	508/254	320/442	713/49	633/48	NA	NA	NA
Kim et al. (40)	1997AJCC	1973 WHO/ ISUP	31/207	131/107	95/143	NA	182/56	185/53	NA	10/228
Margulis et al. (41)	2002 AJCC	1998 WHO/ ISUP	338/1,025	852/511	495/868	455/135	1,341/22	983/380	294/1,069	NA

*NA, data not applicable; AJCC, American Joint Committee on Cancer classification; WHO/ ISUP:World Health Organization/International Society of Urological Pathology classification; LVI, Lymphovascular Invasion; LNM, Lymph node metastasis; TN, Tumor necrosis; PSM, Positive surgical margin*.

### Meta-Analysis Results

**Figure 2:**
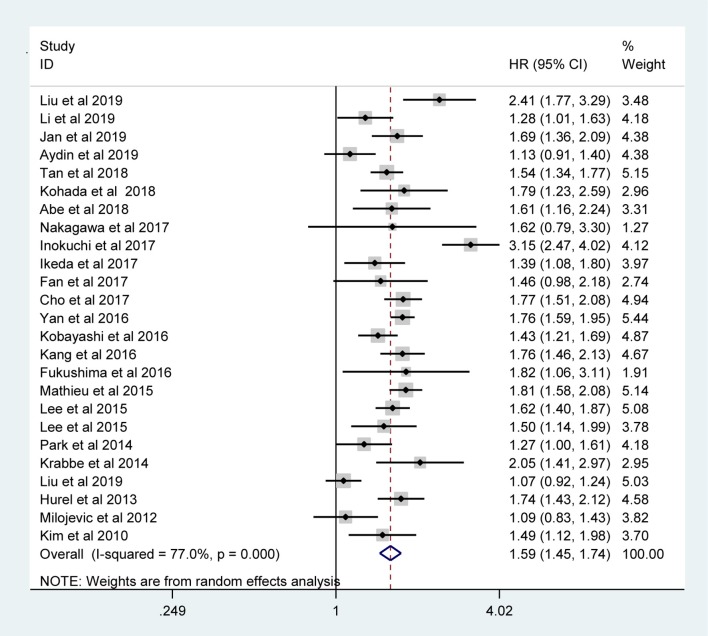
Meta-analysis of the effect of LVI on CSS.

**Figure 3:**
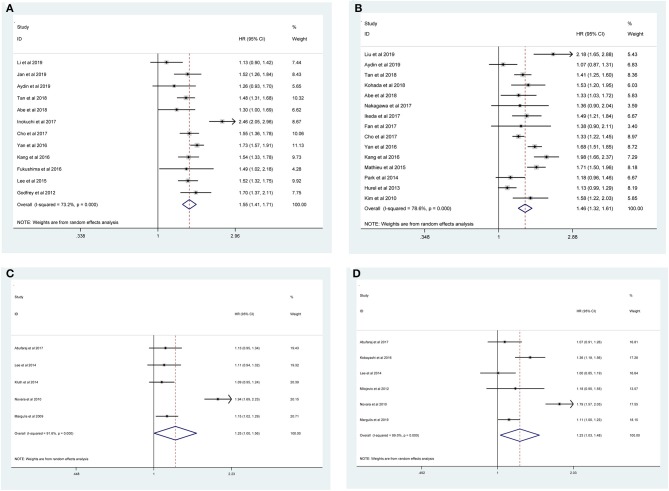
Forest plots assessing the correlation of LVI and **(A)** OS, **(B)** RFS, **(C)** CSM, and **(D)** recurrence in studies considering patients with UTUC.

The pooled results indicated that the presence of LVI in UTUC specimens was associated with poor CSS (RE model, HR = 1.59, 95% CI: 1.45–1.74, *p* < 0.001; *I*^2^ = 77%) (Figure 2), OS (RE model, HR = 1.55, 95 % CI: 1.41–1.71, *p* < 0.001; ***I***^**2**^= 73.2%) (Figure 3A), RFS (RE model, HR = 1.46, 95 % CI: 1.32–1.61, *p* < 0.001; *I*^2^ = 78.6%) (Figure 3B), CSM (RE model, HR=1.25, 95 % CI: 1.00–1.56, *p* = 0.047; *I*^2^ = 91.6%) (Figure 3C), and recurrence (RE model, HR=1.23, 95 % CI: 1.03–1.48, *p* = 0.026; *I*^2^ = 89%) (Figure 3D). To explore the heterogeneity for CSS, OS, and RFS, the prognostic value of LVI was evaluated using subgroup analysis under the geographical region (Asia vs. non-Asian), year of publication (≥2014 vs. <2014), TNM stage (T3+T4 %) (≥40 vs. <40), tumor grade (G2+G3 %) (≥60 vs. <60), number of patients (≥500 vs. <500), and median follow-up (≥ 40 months vs. <40 months) (Table 3). Because of the few cohorts in the CSM and recurrence groups, no subgroup analysis was conducted. The results in the subgroup analysis were consistent with the primary findings, which suggested that LVI was a prognostic factor despite heterogeneity among some groups. Although no significant changes for the interstudy heterogeneity were detected, the observed heterogeneity dropped significantly in some subgroup models, such as the number of patients <500 and Grade (G3+G4 %) ≥60.

**Table 3:** Summary and subgroup analysis of pooled ORs for the eligible studies.

**Analysis specification**	**No. of studies**	**Study heterogeneity**		**Effects model**	**Pooled HR(95% CI)**	***P*-Value**
		**I**^****2****^ **(%)……P**_****heterogeneity****_				
**CSS**						
Overall	24	69.9	<0.001	Random	1.62(1.49,1.76)	<0.001
Geographical region						
Asia	19	65.1	<0.001	Random	1.66(1.52,1.81)	<0.001
non-Asian	5	82.5	<0.001	Random	1.51(1.19,1.91)	0.001
Year of publication						
≥ 2014	16	74	<0.001	Random	1.67(1.49,1.86)	<0.001
<2014	8	59.4	0.016	Random	1.55(1.37,1.76)	<0.001
No. of patients						
≥ 500	9	77.5	<0.001	Random	1.83(1.63,2.06)	<0.001
<500	15	34.8	0.090	Fixed	1.45(1.33,1.59)	<0.001
Stage (T_3_+T_4_ %)						
≥ 40	16	68.8	<0.001	Random	1.64(1.49,1.81)	<0.001
<40	8	74.3	<0.001	Random	1.58(1.34,1.87)	0.001
Grade (G_2_+G_3_ %)						
≥ 60	15	51.6	0.011	Random	1.60(1.48,1.73)	<0.001
<60	9	82.9	<0.001	Random	1.70(1.39,2.07)	<0.001
Median follow-up						
≥ 40 months	14	72.1	<0.001	Random	1.65(1.45,1.88)	<0.001
<40 months	10	69	<0.001	Random	1.59(1.42,1.78)	<0.001
**OS**						
Overall	12	73.2	<0.001	Random	1.55(1.41,1.71)	<0.001
Geographical region						
Asia	10	76.5	<0.001	Random	1.56(1.40,1.74)	<0.001
non-Asian	2	61.0	0.109	Random	1.49(1.11,2.00)	<0.001
Year of publication						
≥ 2014	10	77.6	<0.001	Random	1.54(1.37,1.73)	<0.001
<2014	2	0	0.402	Fixed	1.58(1.40,1.77)	<0.001
No. of patients						
≥ 500	4	83.1	<0.001	Random	1.71(1.48,1.97)	<0.001
<500	8	33.9	0.169	Fixed	1.43(1.29,1.59)	<0.001
Stage (T_3_+T_4_ %)						
≥ 40	10	71.2	<0.001	Random	1.60(1.45,1.78)	<0.001
<40	2	80.1	0.025	Random	1.33(0.99,1.80)	0.061
Grade (G_3_+G_4_ %)						
≥ 60	9	0	0.449	Fixed	1.58(1.50,1.66)	<0.001
<60	3	93.8	<0.001	Random	1.54(0.92,2.58)	0.098
Median follow-up						
≥ 40 months	7	82.6	<0.001	Random	1.54(1.30,1.81)	<0.001
<40 months	5	29.4	0.225	Fixed	1.60(1.47,1.75)	<0.001
**RFS**						
Overall	15	78.6	<0.001	Random	1.46(1.32,1.61)	<0.001
Geographical region						
Asia	12	69.3	<0.001	Random	1.52(1.38,1.67)	<0.001
non-Asian	3	91.8	<0.001	Random	1.28(0.94,1.74)	0.114
Year of publication						
≥ 2014	11	75.8	<0.001	Random	1.50(1.34,1.67)	<0.001
<2014	4	86.4	<0.001	Random	1.38(1.09,1.74)	0.007
No. of patients						
≥ 500	7	88.2	<0.001	Random	1.57(1.36,1.81)	<0.001
<500	8	29.6	0.192	Fixed	1.34(1.20,1.49)	<0.001
Stage (T_3_+T_4_ %)						
≥ 40	11	68.9	0.061	Random	1.57(1.31,1.88)	<0.001
<40	4	82.7	0.026	Random	1.84(0.95,3.53)	0.068
Grade (G_3_+G_4_ %)						
≥ 60	4	45.8	<0.001	Random	1.38(1.25,1.51)	<0.001
<60	2	54.1	0.001	Random	1.71(1.36,2.15)	<0.001
Median follow-up						
≥ 40 months	9	46.8	0.059	Random	1.42(1.30,1.57)	<0.001
<40 months	6	89.2	<0.001	Random	1.48(1.23,1.79)	<0.001

The risk estimate with pooled ORs was used to assess the associations between the LVI and the clinicopathological parameters in patients with UTUC. As shown in Table 4, LVI was significantly related to TNM stage (III/IV vs. I/II: OR = 7.63, 95% CI: 5.60–10.39, *p* < 0.001) (Supplementary Figure 1A), higher tumor grade (3 vs. 1/2: OR = 5.61, 95% CI: 3.71–8.48, *p* < 0.001) (Supplementary Figure 1B), lymph node metastasis (LNM) (yes vs. no: OR = 4.95, 95% CI: 3.66–6.71, *p* < 0.001) (Supplementary Figure 1C), concomitant carcinoma *in situ* (CIS) (yes vs. no: OR = 1.92, 95% CI: 1.36–2.70, *p* < 0.001) (Supplementary Figure 1D), and positive surgical margin (PSM) (yes vs. no: OR = 4.38, 95% CI: 2.71–7.07, *p* < 0.001) (Supplementary Figure 1E), but not related to gender (male vs. female: OR = 0.98, 95% CI: 0.80–1.19, *p* = 0.825) (Supplementary Figure 2A) and multifocality (multifocal vs. unifocal: OR = 1.09, 95% CI: 0.82–1.46, *p* = 0.555) (Supplementary Figure 2B). No significant heterogeneity was observed in those groups. In sensitivity analyses omitting enrolled studies in turn, the results showed that the pooled HRs did not alter significantly, which suggested that the findings were reliable and robust (Supplementary Figure 3).

**Table 4:** Meta-analysis of LVI and clinicopathological features in patients with UTUC.

**Variables**	**Studies**	**Pooled OR (95% CI)**	***P*_**Value**_**	**Model**	**Heterogeneity *I^**2**^* (%)**	***P _***Heterogeneity***_***
TNM stage (III/IV vs. I/II)	7	7.63 (5.60–10.39)	<0.001	RE	44.2	0.097
Tumor grade (3 vs. 1/2)	7	5.61 (3.71–8.48)	<0.001	RE	45.2	0.090
Lymph node metastasis (yes vs. no)	6	4.95 (3.66–6.71)	<0.001	FE	0	0.625
Carcinoma *in situ* (yes vs. no)	4	1.92 (1.36–2.70)	<0.001	FE	0	0.826
Positive surgical margin (yes vs. no)	3	4.38 (2.71–7.07)	<0.001	FE	0	0.794
Multifocality (multifocal vs. unifocal)	6	1.09 (0.82–1.46)	0.555	FE	36.1	0.166
gender (male vs. female)	7	0.98 (0.80–1.19)	0.825	FE	0	0.675

### Publication Bias

**Figure 4:**
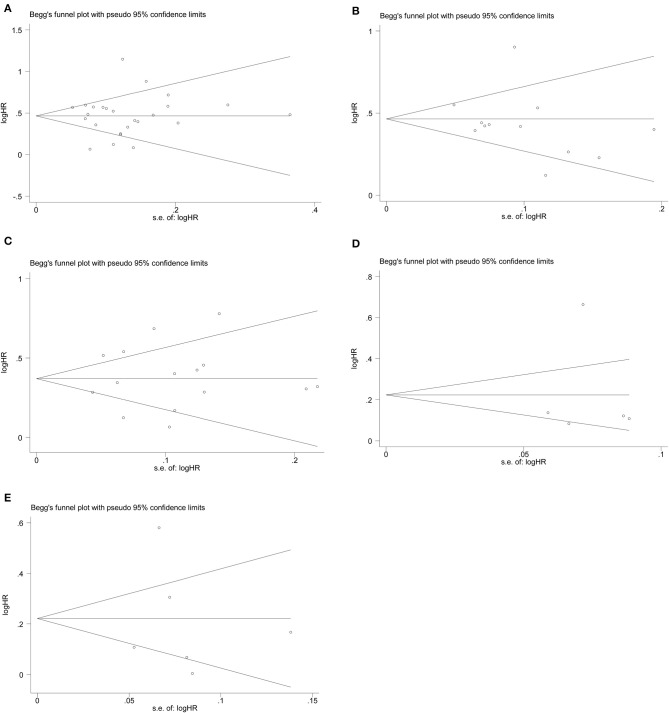
Funnel plots evaluating possible publication bias regarding **(A)** CSS, **(B)** OS, **(C)** RFS, **(D)** CSM, and **(E)** recurrence.

We conducted the publication bias assessment of the studies using funnel plots and Egger's test. As shown in Figure 4, no obvious asymmetry was observed in all of the groups. The *P* values of the Egger's test were all >0.05 in CSS (*p*-Egger = 0.977) (Figure 4A), OS (*p*-Egger = 0.330) (Figure 4B), RFS (*p*-Egger = 0.811) (Figure 4C), CSM (*p*-Egger = 0.984) (Figure 4D), and recurrence (*p*-Egger = 0.843) (Figure 4E).

## Discussion

UTUC is a rare urothelial carcinoma compared with bladder cancer; however, the incidence of UTUC has increased significantly in the last decade (42). Although we have made great strides to understand UTUC, its management remains challenging. Even after the standard RNU surgery was performed in the majority of patients with UTUC, there were still some patients with poor postoperative outcomes. Therefore, it is very important to accurately predict the clinical prognosis of patients with UTUC after RNU. To date, many studies have been conducted to identify the significant prognostic factors of UTUC. Several traditional prognostic predictors, such as pathologic characteristics of RNU specimens including tumor stage and grade (40), tumor architecture (21), tumor size (24), and CIS (43), have been identified as significant prognostic variables for CSS and RFS in different studies.

LVI was considered to be the first step in the metastasis of tumor cells and LNM (30). Recently, mounting evidence has indicated that LVI is associated with poor prognosis for many types of tumors, such as liver, bladder, and prostate cancer (7, 44). Jiang et al. (6) reported that LVI is an independent prognostic factor for predicting worse progression in prostate cancer, and they recommended that LVI should be reported in the final pathological diagnosis after radical prostatectomy. Similarly, Canter et al. (45) found that the presence of LVI in the final pathological reports for bladder cancer delivers significant risks for worse CSS and OS. Several studies have suggested that LVI can be used as an independent prognostic factor in patients with UTUC after RNU (29, 36). However, some studies have suggested that the prognostic value of LVI in assessing survival outcome is meaningless (28, 46). The possible outcomes of these few negative papers may have been related to the heterogeneity of UTUC biology and different clinicopathological features.

Based on the findings of previous research, ~15%−30% of patients with UTUC after surgery have a positive rate of LVI in the final pathology report (8, 30, 39). Consistent with results in previous reports, we found that LVI appeared in ~24.8% of patients. LVI is an easily accessible pathological parameter, which can be accurately measured among observers. Hurel et al. (36), in their study involving 551 patients, concluded that the presence of LVI was an independent risk factor for UTUC. Likewise, Lee et al. (30) reported a significant association between LVI and tumor grade, tumor stage, and LNM. Although it has been proposed that LVI should be accurately recorded in the pathological reports for UTUC specimens, there are still controversial data regarding the impact of LVI on patient prognosis and survival. For example, Jan et al. (13) recently reported that LVI was not associated with OS and CSS in a multivariate analysis. Eich et al. (47) found that LVI was not associated with tumor progression, total mortality, and CSM.

Although the previous studies had largely enhanced our knowledge of UTUC, they were limited to small sample sizes and heterogeneous populations. To overcome these shortcomings and better understand the clinical value of LVI, we assessed LVI and 14,653 patients treated with RNU for UTUC using a meta-analysis. In this study, we demonstrated that LVI was an independent prognostic factor for CSS, RFS, OS, CSM, and recurrence among patients with UTUC treated with RNU. In several studies, LVI has been related to worse tumor differentiation, higher stage and grade, LNM, and PSMs. Consistent with findings in previous outcomes (11, 30, 36), our results indicated that LVI was associated with clinicopathological features, which are all independent poor prognostic factors. All of the results strongly supported the prognostic value of LVI with regard to poor outcomes in UTUC and its role in tumor progression. Furthermore, the results of the study may provide a postoperative follow-up protocol for patients with UTUC to evaluate the prognostic value of LVI. Interestingly, no obvious association between LVI, and multifocality was found in our study. Although multifocal tumors had a worse oncological outcome than renal pelvic tumors, the role of tumor location has not yet been confirmed (39, 48). Hence, multifocal tumors may develop from a more aggressive carcinogenesis pathway.

The results obtained in this study are mainly consistent with the outcomes in a previous system review by Ku et al. (8). However, our study presented a series of advancements. At first, the search time by Ku et al. ended in 2013. However, we added 23 extra studies including 10,963 patients, thereby allowing us to perform a subgroup analysis with more exact evaluation for LVI. Besides, the quality as assessed by NOS in the present meta-analysis was greater, which strengthened the persuasiveness of this research. With the stupendous prognostic value of LVI, we suggest that LVI should always be presented in the pathologic report of RNU specimens. Moreover, patients with LVI expression may be given intensive treatment after RNU. Currently, there is insufficient evidence to recommend adjuvant chemotherapy (AC) as a treatment strategy for patients with UTUC (49). Lee et al. (29) showed that AC does not reduce mortality in patients with UTUC. However, in the subgroup of patients with LVI, AC could significantly improve CSS and OS. Unfortunately, we are unable to further explore the relationship between LVI and AC due to the insufficient data in this study.

Our study has several limitations that should be acknowledged. First, the literature was mainly retrospective, with an obvious heterogeneity in our study. A subgroup analysis that aimed to identify the source of heterogeneity was conducted in the present study. Although considerable heterogeneity among studies had no effect on the pooled results, heterogeneity should not be completely neglected. Thus, the conclusions yielded in this study must be interpreted with caution. Second, the studies in our paper were mainly conducted in four regions. The observed differences in the statistical results might reflect regional ethnic differences. Third, other potential risk factors involved in this report may have affected the final results. For example, the surgical methods were different. Most RNUs were laparoscopic approaches, but some were performed by open surgeries. Thus, there may be performance bias. Fourth, reporting bias may exist in our research, as some studies with negative results may not have been published. However, no significant bias was observed in this research, which indicates that our results were stable and reliable.

## Conclusion

In summary, LVI is associated with unfavorable prognosis and clinicopathological features in patients with UTUC. Given its convenience and inexpensiveness in clinical application, LVI could be a useful tool for predicting prognosis and outcomes of patients with UTUC. However, additional prospective, multicenter studies should be conducted to confirm our findings and address the limitations observed in our meta-analysis.

## Data Availability Statement

All data generated or analyzed during this study are included in this published article.

## Author Contributions

LZ project development and manuscript writing. BW data management and manuscript editing. JY and YF data collection. ZZ and HZ data analysis and data management.

## Conflict of Interest

The authors declare that the research was conducted in the absence of any commercial or financial relationships that could be construed as a potential conflict of interest.

## Supplementary Material

Supplementary Figure 1 Forest plots of meta-analyses of the association between LVI and clinicopathological features in UTUC: **(A)** TNM stage, **(B)** tumor grade, **(C)** LNM, **(D)** CIS, and **(E)** PSM.

Supplementary Figure 2 Forest plots of meta-analyses of the association between LVI and clinicopathological features in UTUC: **(A)** gender and **(B)** multifocality.

Supplementary Figure 3 Sensitivity analysis in this meta-analysis. **(A)** Sensitivity analysis for CSS; **(B)** sensitivity analysis for OS; **(C)** sensitivity analysis for RFS; **(D)** sensitivity analysis for CSM; and **(E)** sensitivity analysis for recurrence.

## Abbreviations

LVI: lymphovascular invasion
UTUC: upper tract urinary carcinoma
RNU: radical nephroureterectomy
PRISMA: Preferred Reporting Items for Systematic Reviews and Meta-Analyses
NOS: Newcastle-Ottawa scale
HRs: hazard ratios
ORs: odds ratio
CIs: confidence intervals
CSS: cancer-specific survival
OS: overall survival
RFS: recurrence-free survival
CSM: cancer-specific mortality
CIS: concomitant carcinoma *in situ*
LNM: lymph node metastasis
PSM: positive surgical margin
AC: adjuvant chemotherapy.
