# Supplementary figures and images for: The Prognostic Value of Lymphovascular Invasion in Patients With Upper Tract Urinary Carcinoma After Surgery: An Updated Systematic Review and Meta-Analysis
Source: Front Oncol. 2020 Apr 22;10:487. doi: 10.3389/fonc.2020.00487 (PMC7189418; doi:10.3389/fonc.2020.00487)

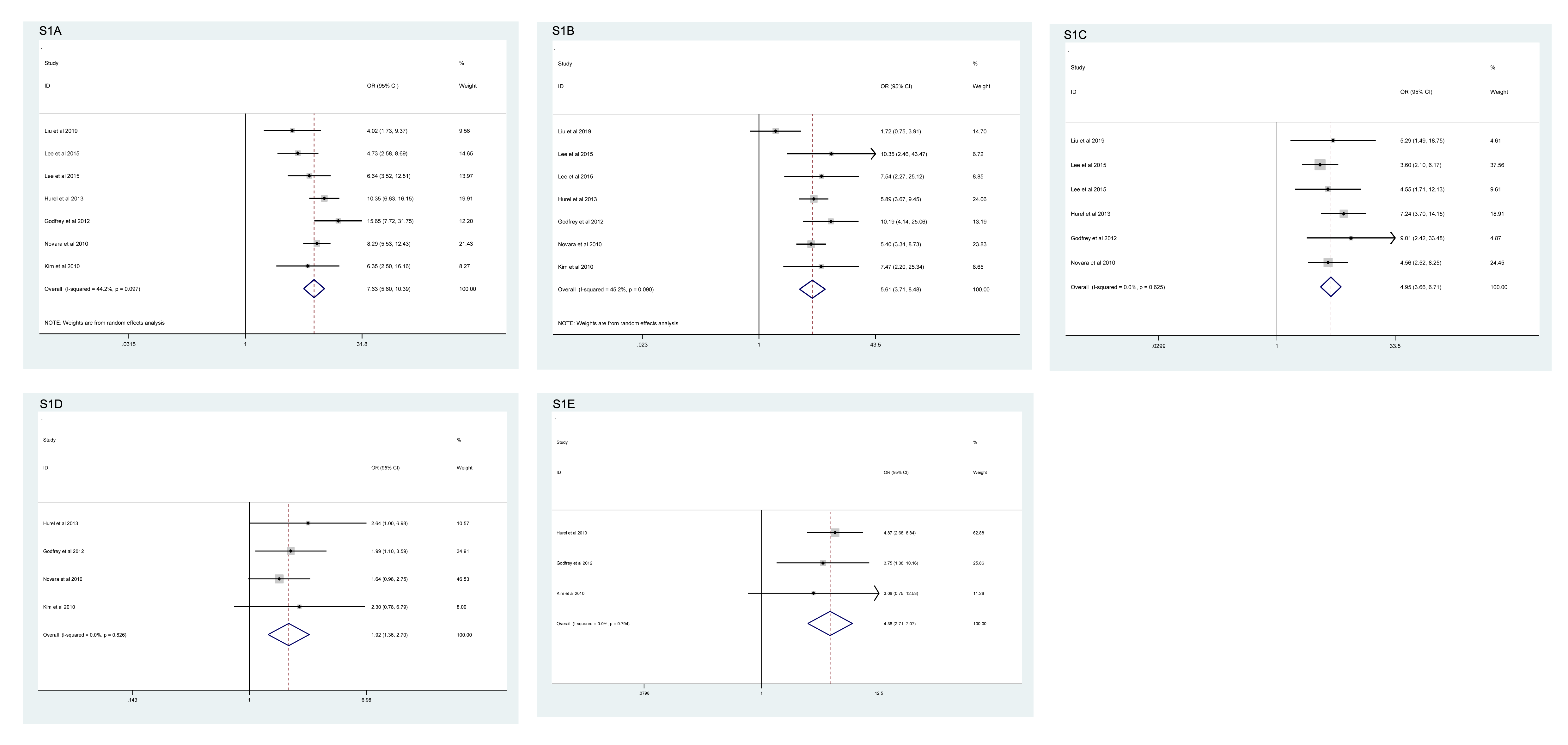

Supplement: Supplementary Figure 1 — Forest plots of meta-analyses of the association between LVI and clinicopathological features in UTUC: (A) TNM stage, (B) tumor grade, (C) LNM, (D) CIS, and (E) PSM. [file Image_1.TIF]

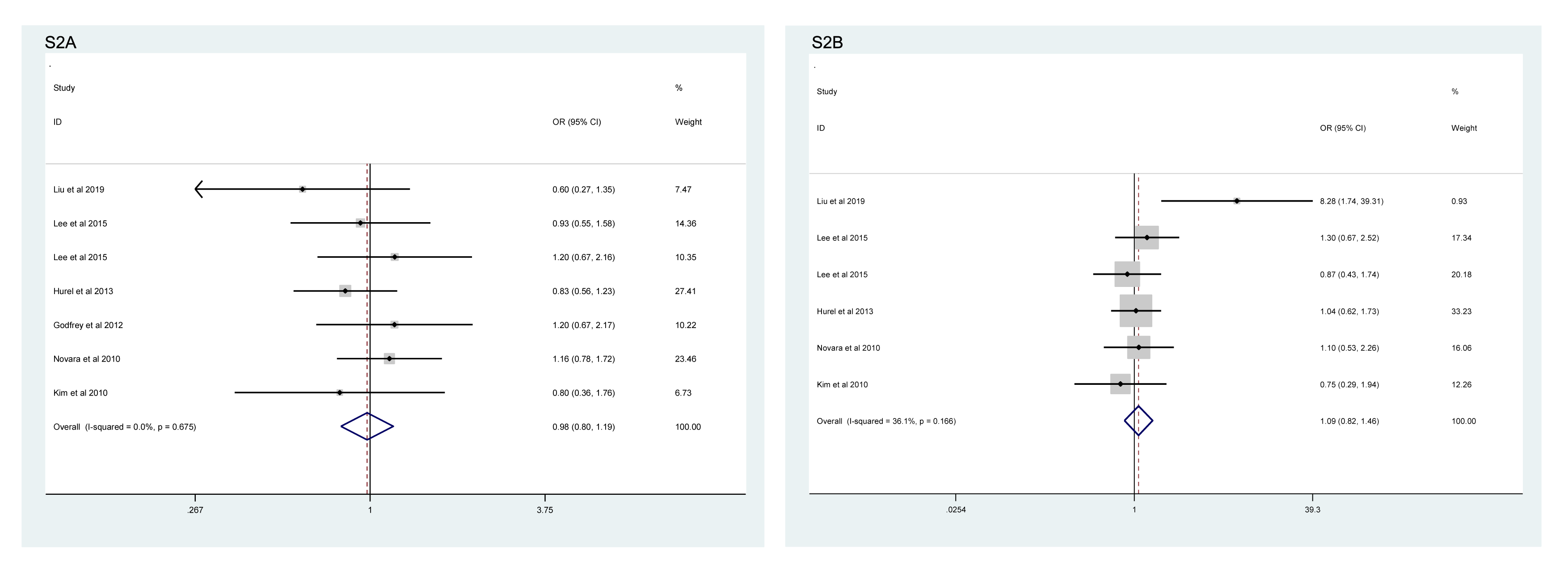

Supplement: Supplementary Figure 2 — Forest plots of meta-analyses of the association between LVI and clinicopathological features in UTUC: (A) gender and (B) multifocality. [file Image_2.TIF]

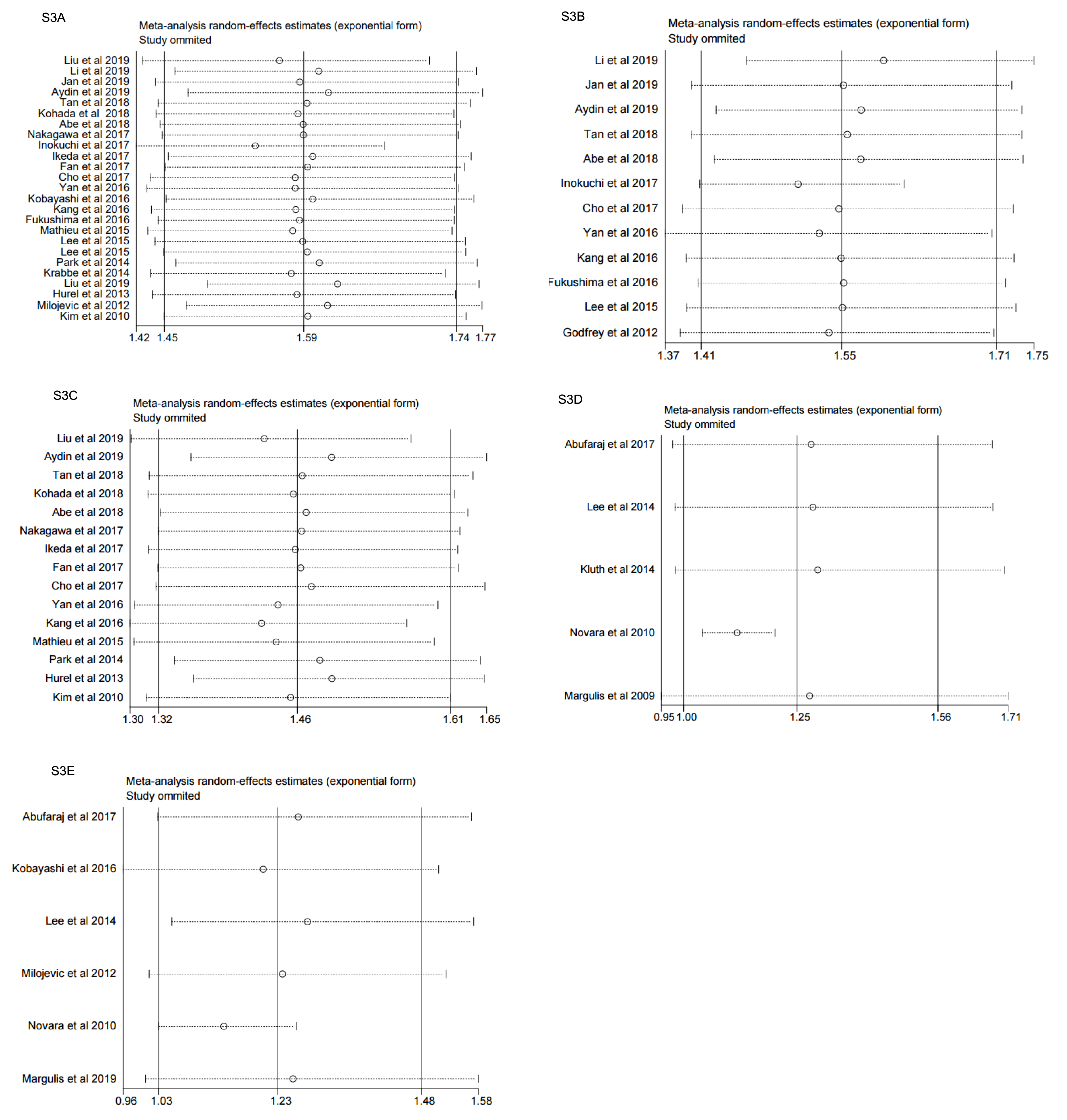

Supplement: Supplementary Figure 3 — Sensitivity analysis in this meta-analysis. (A) Sensitivity analysis for CSS; (B) sensitivity analysis for OS; (C) sensitivity analysis for RFS; (D) sensitivity analysis for CSM; and (E) sensitivity analysis for recurrence. [file Image_3.TIF]
