# Supplementary material for: The Prognostic Value of Lymphovascular Invasion in Patients With Upper Tract Urinary Carcinoma After Surgery: An Updated Systematic Review and Meta-Analysis
Source: Front Oncol. 2020 Apr 22;10:487. doi: 10.3389/fonc.2020.00487 (PMC7189418; doi:10.3389/fonc.2020.00487)
Supplement: Supplementary file 4 [file Table_1.docx]

**Supplementary Table 1.** Quality assessment of the included studies in this meta- analysis

| **Study** | **Representativeness of the exposed cohort** | **Selection of the unexposed cohort** | **Ascertainment of exposure** | **Outcome of interest not present at start of study** | **Control for important factor or additional factor** | **Outcome assessment** | **Follow-up long enough for outcomes to occur** | **Adequacy of follow-up of cohort** | **Total quality scores** |
| --- | --- | --- | --- | --- | --- | --- | --- | --- | --- |
| Liu et al[[11](#_ENREF_11)] | ★ | ★ | ★ | ★ | ★★ | ★ | ★ | ★ | 9 |
| Li et al[[12](#_ENREF_12)] | ★ | ★ | ★ | ★ | ★★ | ★ | ★ | ★ | 9 |
| Jan et al[[13](#_ENREF_13)] | ★ | ★ | ★ | ★ | ★★ | ★ | ★ | ★ | 9 |
| Aydin et al[[14](#_ENREF_14)] | ★ | ★ | ★ | ★ | ★ | ★ | ★ | ★ | 8 |
| Tan et al[[15](#_ENREF_15)] | ★ | ★ | ★ | ★ | ★★ | ★ | ★ | ★ | 9 |
| Kohada et al[[16](#_ENREF_16)] | ★ | ★ | ★ | ★ | ★★ | ★ | ★ | ★ | 9 |
| Abe et al[[17](#_ENREF_17)] | ★ | ★ | ★ | ★ | ★★ | ★ | ★ | ★ | 8 |
| Nakagawa et al[[18](#_ENREF_18)] | ★ | ★ | ★ | ★ | ★★ | ★ | ★ | ★ | 9 |
| Inokuchi et al[[19](#_ENREF_19)] | ★ | ★ | ★ | ★ | ★★ | ★ | ★ | ★ | 9 |
| Ikeda et al[[20](#_ENREF_20)] | ★ | ★ | ★ | ★ | ★★ | ★ | ★ | ★ | 9 |
| Fan et al[[21](#_ENREF_21)] | ★ | ★ | ★ | ★ | ★★ | ★ | ★ | ★ | 9 |
| Cho et al[[22](#_ENREF_22)] | ★ | ★ | ★ | ★ | ★★ | ★ | ★ | ★ | 9 |
| Abufaraj et al[[23](#_ENREF_23)] | ★ | ★ | ★ | ★ | ★ | ★ | ★ | ★ | 8 |
| Yan et al[[24](#_ENREF_24)] | ★ | ★ | ★ | ★ | ★★ | ★ | ★ | ★ | 9 |
| Kobayashi et al[[25](#_ENREF_25)] | ★ | ★ | ★ | ★ | ★★ | ★ | ★ | ★ | 8 |
| Kang et al[[26](#_ENREF_26)] | ★ | ★ | ★ | ★ | ★★ | ★ | ★ | ★ | 9 |
| Fukushima et al[[27](#_ENREF_27)] | ★ | ★ | ★ | ★ | ★ | ★ | ★ | ★ | 8 |
| Mathieu et al[[28](#_ENREF_28)] | ★ | ★ | ★ | ★ | ★★ | ★ | ★ | ★ | 9 |
| Lee et al[[29](#_ENREF_29)] | ★ | ★ | ★ | ★ | ★★ | ★ | ★ | ★ | 9 |
| Lee et al[[30](#_ENREF_30)] | ★ | ★ | ★ | ★ | ★★ | ★ | ★ | ★ | 9 |
| Park et al[[31](#_ENREF_31)] | ★ | ★ | ★ | ★ | ★★ | ★ | ★ | ★ | 9 |
| Lee et al[[32](#_ENREF_32)] | ★ | ★ | ★ | ★ | ★ | ★ | ★ | ★ | 8 |
| Krabbe et al[[33](#_ENREF_33)] | ★ | ★ | ★ | ★ | ★★ | ★ | ★ | ★ | 9 |
| Kluth et al[[34](#_ENREF_34)] | ★ | ★ | ★ | ★ | ★ | ★ | ★ | ★ | 8 |
| Liu et al[[35](#_ENREF_35)] | ★ | ★ | ★ | ★ | ★ | ★ | — | ★ | 7 |
| Hurel et al[[36](#_ENREF_36)] | ★ | ★ | ★ | ★ | ★★ | ★ | ★ | ★ | 9 |
| Milojevic et al[[37](#_ENREF_37)] | ★ | ★ | ★ | ★ | ★ | ★ | ★ | ★ | 8 |
| Godfrey et al[[38](#_ENREF_38)] | ★ | ★ | ★ | ★ | ★★ | ★ | ★ | ★ | 9 |
| Novara et al[[39](#_ENREF_39)] | ★ | ★ | ★ | ★ | ★★ | ★ | ★ | ★ | 9 |
| Kim et al[[40](#_ENREF_40)] | ★ | ★ | ★ | ★ | ★★ | ★ | ★ | ★ | 9 |
| Margulis et al[[41](#_ENREF_41)] | ★ | ★ | ★ | ★ | ★★ | ★ | ★ | ★ | 9 |
